# Just What Should Have Been Done

**Published:** 1901-06

**Authors:** 


### JUST WHAT SHOULD HAVE BEEN DONE.

THE reappointment by Governor Stone, of Pennsylvania, of Dr. Leonard Pearson as State Veterinarian is just what should have been done.

The record of Dr. Pearson in State veterinary sanitary work stands foremost to-day in the union of States of our country as one the profession may well be proud of. The excellent work done for the well-being of our people and the results attained in the Keystone State have won the approval of the whole people, and the losses from which those engaged in animal industry have been saved are enormous in the aggregate. The veterinary profession has not been without his aid in every direction, and much added strength has followed his incumbency of this office, until the State to-day has practically a sanitary cordon at the least possible cost for such much-needed service.

